# Can Zoledronic Acid be Beneficial for Promoting Tumor Response in Breast Cancer Patients Treated with Neoadjuvant Chemotherapy?

**DOI:** 10.3390/jcm2040188

**Published:** 2013-10-16

**Authors:** Ayoub Charehbili, Duveken B. Y. Fontein, Judith R. Kroep, Gerrit-Jan Liefers, Johannes W. R. Nortier, Cornelis J. H. van de Velde

**Affiliations:** 1 Department of Surgery, Leiden University Medical Center, Albinusdreef 2, ZA Leiden 2333, The Netherlands; E-Mails: d.b.y.fontein@lumc.nl (D.B.Y.F.); g.j.liefers@lumc.nl (G.-J.L.); c.j.h.van_de_velde@lumc.nl (C.J.H.V.); 2 Department of Medical Oncology, Leiden University Medical Center, Albinusdreef 2, ZA Leiden 2333, The Netherlands; E-Mails: j.r.kroep@lumc.nl (J.R.K.); j.w.r.nortier@lumc.nl (J.W.R.N.)

**Keywords:** bisphosphonates, zoledronic acid, chemotherapy, neoadjuvant, postmenopausal

## Abstract

The antitumor effect of bisphosphonates (BPs) is under increasing scrutiny. Preclinical and clinical evidence has shown that BPs might sensitize breast tumors to chemotherapy. Here, we present a review of current preclinical and clinical evidence for antitumor effects of BPs, and evaluate how BPs might play a role in neoadjuvant treatment of women with breast cancer.

## 1. Introduction

Neoadjuvant chemotherapy (NCT) is a generally accepted and worldwide standardized treatment for patients with locally advanced or large operable (stage II–III) breast cancer [1]. NCT is as effective as adjuvant chemotherapy following local treatment in terms of (recurrence-free) survival [2]. Besides the opportunity to study changes in tumor biology and response, NCT has the capability of downstaging breast tumors, facilitating in breast conserving surgery. The antitumor effect of adding bisphosphonates (BPs) to (neo)adjuvant chemotherapy for breast cancer is still under debate. There is emerging preclinical evidence for a synergistic effect of the most potent BP, zoledronic acid, in combination with chemotherapy, when administered after chemotherapy [3]. Clinical results suggest that BPs might improve treatment efficacy in patients with breast cancer [4,5]. Clinically, menopausal and/or hormonal status seem to play a role. The neoadjuvant model is ideal for gaining insight into the biological antitumor mechanisms of BPs in combination with NCT and can aid in defining predictors of response for this treatment strategy. Here, we provide a comprehensive review of preclinical and clinical evidence for the antitumor effects of BPs and a rationale for possible efficacy of BPs in the neoadjuvant setting.

## 2. Bisphosphonates

BPs are pyrophosphates and can be divided into two subgroups based on the structure of the R2 side chain: non-nitrogen containing BPs (e.g., clodronate), and the more potent nitrogen-containing BPs (e.g., zolendronic acid, alendronate, ibandronate, risedronate), which are widely used in the clinical setting [6]. Zoledronic acid is currently the most potent available BP containing two nitrogen atoms [7,8,9]. 

**Figure 1:**
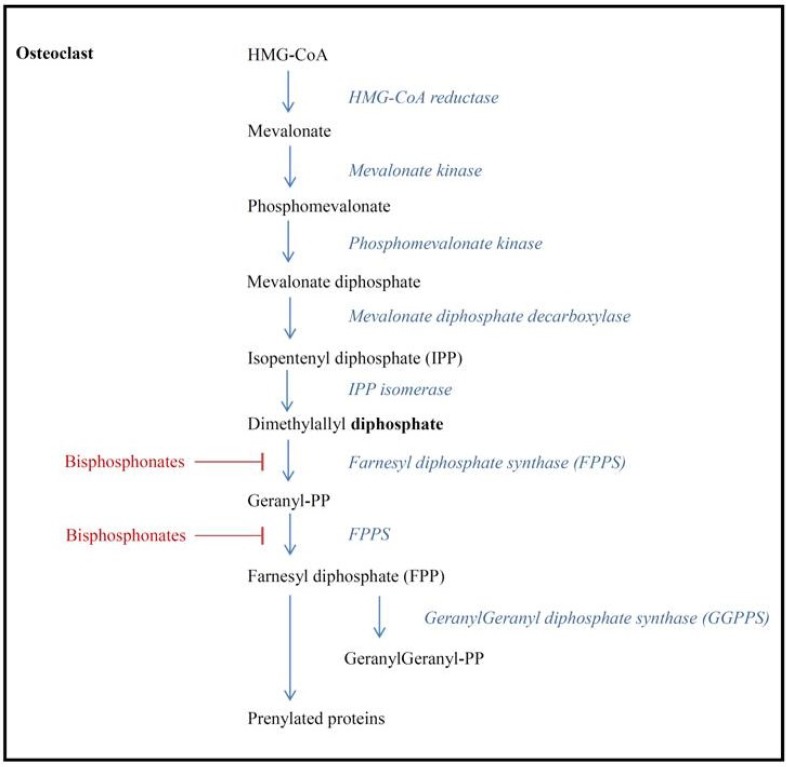
Mevalonate pathway and inhibition of farnesyl diphosphate synthase by bisphosphonates.

As all pyrophosphates, BPs easily bind to the bone mineral with the P–C–P chain at locations showing a high level of bone resorption. BPs inhibit the breakdown of hydroxyapatite, thereby suppressing bone resorption and promoting osteoclast apoptosis [10]. Nitrogen-containing BPs bind to and inhibit farnesyl pyrophosphate synthase (FPPS), which is an important regulatory enzyme of the mevalonate pathway (Figure 1), and which is responsible for the production of lipids needed for the posttranslational modification (prenylation) of proteins and activation of intracellular signaling proteins [11,12,13]. These signaling proteins are essential for cell functioning and survival, and osteoclast apoptosis is induced by inhibiting the posttranslational modification of proteins with isoprenyl [13]. Furthermore, nitrogen-containing BPs induce the production of an adenosine triphosphate analogue (Apppi) that can directly induce apoptosis [14]. In addition, bisphosphonates have been found to inhibit both osteoblast and osteocyte apoptosis [15].

BPs have a well-established role in the prevention and treatment of osteoporosis and in the treatment of bone metastases, causing a reduction in pain, hypercalcemia of malignancy and skeletal related events (SRE), such as pain, pathological fractures, and spinal cord compression. These SREs are a major cause of morbidity and a reduced quality of life [7]. In addition to their use in treating osteoporosis and bone metastases, bisphosphonates are gaining recognition for the management of breast cancer through various mechanisms, and their use has grown rapidly in recent years. Needless to say, the mechanisms by which BPs prevent and decrease tumor burden in bone is currently still speculative and under investigation.

## 3. Hypotheses for BP Anti-Tumor Mechanism

There is ample evidence to suggest that the mechanism of bone metastases is multifaceted, comprising both bone resorption and bone formation aided by osteoblast and osteoclast activity [16]. In breast cancer, bone metastases are generally characterized by a predominantly osteoclastic activity, with osteolysis the result of osteoclast stimulation. In response, there is some degree of bone formation or bone repair, caused by osteoblasts [16].

Cancer cells produce a range of growth factors and cytokines that increase osteoclast activity [17]. Tumor production of factors including parathyroid hormone (PTH), PTH-related peptide (PTHrP) and interleukins (IL)-1, IL-6 and IL-11 stimulate the production of the cytokine, receptor activator of nuclear factor-KB ligand (RANKL), by osteoblasts and stromal cells. Following stimulation by PTHrP, RANKL induces osteoclast activity. PTHrP also causes a decrease in the production of osteoprotegrin (OPG), a receptor that prevents RANKL from binding to its receptor (RANK) on osteoclast progenitor cells, thereby blocking bone resorption [16].

**Figure 2:**
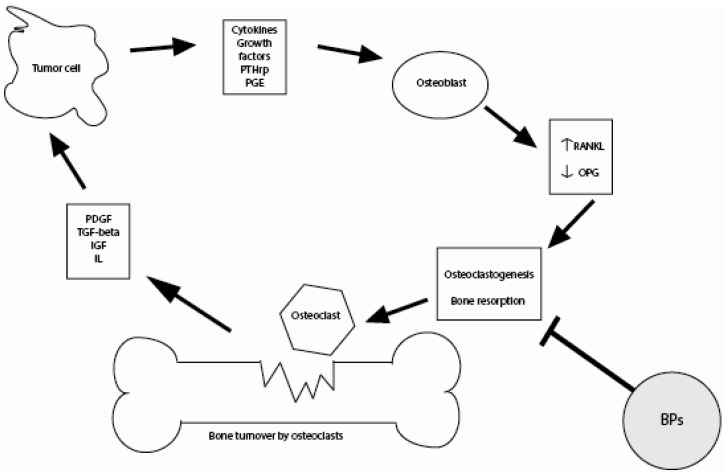
Schematic diagram of the interaction between the bone microenvironment and tumor cells.

During bone resorption, other potentially tumor-stimulating growth factors such as transforming growth factorbeta (TGF-beta) and insulin-like growth-factor-1 are released by osteoblasts, facilitating tumor cell growth and proliferation, and attracting other tumor cells [17]. BPs may reduce tumor burden and growth by inhibiting this bone turnover. BPs do this, both directly, through the apoptosis of osteoclasts and tumor cells, and indirectly, through alterations in the bone microenvironment (Figure 2). Direct effects include the metabolism of non-nitrogen-containing BPs to an adenosine triphosphate analog that is toxic for macrophages and osteoclasts [14]. Nitrogen-containing BPs also work through several indirect mechanisms. For example, BPs may render the bone microenvironment less favorable for tumor cell growth. Namely, in case of skeletal metastases, a balanced coupling of osteoblastic bone formation and osteoclastic bone resorption is lost [18]. BPs can interrupt this vicious cycle of osteolytic bone loss.

In addition, BPs inhibit angiogenesis, as demonstrated in one study, where zoledronic acid was found to reduce circulating levels of vascular endothelial growth factor (VEGF) after the first infusion in patients with metastatic bone disease [19,20]. Lastly, and most importantly in the neoadjuvant setting, BPs may reduce tumor burden by indirectly modulating the immune system. For example, BPs enhance cellular antitumor toxicity by attracting and triggering expression of γ/δ T-cells, which could be an important factor in antigen specificity and the ability to recognize and kill tumor cells [21,22,23]. Furthermore, bisphosphonates are suggested to differentiate monocytes into tumoricidal M1 macrophages [24].

Tumor cells produce cytokines and growth factors which stimulate osteoclastogenesis, inducing an increase in bone resorption and the release of growth factors from the bone matrix. These factors stimulate proliferation, migration and angiogenesis of tumor cells. Bisphosphonates can break this vicious circle by inhibiting osteoclastogenesis and bone resorption. PTHrp = parathormone-related protein; PGE = prostaglandin E; RANKL = receptor activator of nuclear factor κβ ligand; OPG = osteoprotegerin; PDGF = platelet derived growth factor; IGF = insulin-like growth factor; IL = interleukin.

Needless to say, there is still a need for more translational research giving insight into the alleged anti-tumor effect of bisphosphonates, and further investigations on the role of BPs are most certainly warranted. The neoadjuvant setting provides a suitable platform for this kind of research.

## 4. Preclinical Treatment Efficacy Data

Of particular interest is the potential for BPs to enhance the anti-tumor activity of cytotoxic agents in the context of (neo)adjuvant chemotherapy. *In vitro* data have shown that clinically relevant concentrations of doxorubicin followed by zoledronic acid consistently induced sequence-dependent synergistic apoptosis of cancer cells across several malignant cell lines [25]. However, the drugs alone, in the reverse sequence, and even given synchronously, had little or no effect on apoptosis [21]. In a mouse model, sequence-dependent synergy between doxorubicin and zoledronic acid was observed with complete inhibition of tumor growth associated with enhanced apoptosis and reduced proliferation and angiogenesis. These effects were statistically more pronounced when the zoledronic acid was administered 24 h after chemotherapy, suggesting that an initial priming of tumor cells by doxorubicin renders them more sensitive to subsequent exposure to zoledronic acid [26]. Possible molecular pathways by which sequential treatment with zoledronic acid and doxorubicin induce tumor cell apoptosis and inhibit proliferation were also shown in an *in vivo* model of breast tumor growth in the bone [27]. Interestingly, zoledronic acid specifically inhibited the development of bone metastases in an ovariectomy-induced/postmenopausal mouse model [28].

## 5. Clinical Evidence

Following previous discordant data with the less potent bisphosphonate, clodronate, in the adjuvant setting [29,30,31,32], the Austrian Breast Cancer Study Group-12 trial was the first adjuvant clinical trial to notice an improvement in disease-free survival (DFS), a reduction in distant (non-bone) metastases, locoregional and contralateral relapses, as well as a trend to reduced risk of death, with zoledronic acid (4 mg intraveneously every 6 months for 3 years) added to endocrine treatment with ovarian suppression in premenopausal breast cancer patients [33,34]. The protective effect of zoledronic acid persisted even after a median follow-up of 76 months, with zoledronic-acid-treated patients having a significant reduction in the risk of DFS events (27%) and a significant reduction in the risk of death (41%) when compared with controls [35]. Of note, all patients received goserelin, and were therefore postmenopausal, from an endocrionlogical viewpoint. This has probably contributed largely to the significant benefit of zoledronic acid in these patients. 

Three other similarly designed trials investigated the effect of delayed *vs.* upfront zoledronic acid on bone mineral density in postmenopausal breast cancer patients, with disease recurrence as a secondary endpoint (Z-FAST, E-ZO-FAST and ZO-FAST) [36,37,38]. Fewer DFS events with upfront zoledronic acid were only observed in the ZO-FAST study (37% RR, *p* = 0.05). Based on exploratory analyses, initiating zoledronic acid may have significant DFS benefits. In the AZURE trial, patients were randomized to standard therapy (any (neo)adjuvant chemotherapy and/or endocrine therapy), with or without zoledronic acid during 3 years. At a median follow-up of 59 months, no significant differences in DFS were found in the complete study population [39]. However, when concentrating on the subset of postmenopausal women, a statistically significant difference in DFS was found between the treatment groups (HR 0.74, *p* = 0.04). These results in postmenopausal women are consistent with the findings in premenopausal women in ABSCG-12 trial, suggesting that efficacy of zoledronic acid treatment is dependent on menopausal status and/or hormonal levels. Recently, this was confirmed in a meta-analysis of phase III studies by Yan *et al.* in which treatment with zoledronic acid did not improve DFS in breast cancer patients [5]. However, in the postmenopausal group, a significant benefit in terms of DFS (RR 0.75) distant (RR 0.74) and locoregional recurrence (RR 0.51), was found. Different results were found in a meta-analysis by Valachis *et al*. in which phase II studies were also included [4]. In this study, in which no specific analyses for postmenopausal women were done, zoledronic acid use resulted in a significantly better OS (HR 0.81) in patients with early-stage breast cancer, strengthening the argument for an antitumor effect of zoledronic acid.

## 6. The Future of Bisphosphonates and Neoadjuvant Therapy

In a retrospective subset evaluation of patients in the AZURE trial, adding the BP, zoledronic acid, to neoadjuvant chemotherapy resulted in better tumor shrinkage and a doubling of the pathological complete response rate [40]. Patients who were treated with neoadjuvant chemotherapy received zoledronic acid 6 times every 3 or 4 weeks, depending on their chemotherapeutic schedule. Zoledronic acid also seemed to sensitize the tumor to the effects of neoadjuvant chemotherapy, as the pathological complete response rate was nearly doubled. The preliminary results of the AZURE trial have motivated investigators to investigate the possible benefit of zoledronic acid in the neoadjuvant setting. For example, our study group aimed to determine the pathological response of neoadjuvant chemotherapy, with and without zoledronic acid, in the NEOZOTAC trial. Here, HER2-receptor negative patients with stage II or III breast cancer are treated with 6 three weekly cycles TAC (docetaxel, doxorubicin, cyclophosphamide with pegfilgrastim), with or without zoledronic acid 4 mg intravenously administered within 24 h of the start of each cycle. The toxicity data of this study, showing that there is no significant difference in toxicity between the treatment arms, has recently been presented [41]. Biomarker data from biopsies and surgical specimens, as well as blood sera are currently being collected for translational research. Response results from several other neoadjuvant chemotherapy trials are expected soon (Table 1). In a study by Chavez-Macgregor *et al.* in which patients who were treated with neoadjuvant chemotherapy were retrospectively identified for pCR rate evaluation, 39 patients received bisphosphonates [42]. The pCR rate was higher in the bisphosphonate group than in the non-bisphosponate group, although not statistically significant (25.4% *vs.* 16%, *p* = 0.11). Furthermore, the JONIE-1 group recently presented data of their phase III trial comparing neoadjuvant chemotherapy with and without zoledronic acid [43,44]. Interestingly these results not only suggested that postmenopausal women benefit more from zoledronic acid therapy (18.4% *vs.* 5.1%, *p* = 0.07), but also that triple-negative bisphosphonate-treated patients respond better than their chemotherapy-only counterpart (35.3% *vs.* 11.8%, *p* = 0.06). Aft *et al.* reported a study in which 120 patients were allocated to a neoadjuvant/adjuvant chemotherapy schedule (with four cycles of neoadjuvant epirubicin plus docetaxel and two cycles of adjuvant epirubicin and docetaxel) with no zoledronic acid or zoledronic acid (4 mg i.v.) every 3 weeks, for 1 year [45]. The primary endpoint was the number of patients with detectable disseminated tumor cells (DTCs) at 3 months. Less DTCs were detected in the zoledronic acid group, suggesting that neoadjuvant treatment with zoledronic acid might affect long-term outcome by preventing metastasis. However no significant difference in pathologic complete response was found (22% in the zoledronic acid arm *vs.* 16% in the control arm, *p* = 0.63), although more pathologic complete response was observed in estrogen receptor (ER)-negative/HER2-negative patients (29% in the zoledronic acid arm *vs.* 11% in the control arm). Interestingly, at 5-year follow up, significantly less death and recurrence events occurred among patients with estrogen receptor-negative tumours, which was not observed in the total study group. Neoadjuvant treatment might therefore indeed have a beneficial effect on long-term outcome [45].

As previously mentioned, neoadjuvant studies are valuable for translational research. An example of this in the context of zoledronic acid treatment is the ANZAC study [46]. In this study 40 patients were randomized to neoadjuvant chemotherapy with or without a single infusion of zoledronic acid after the first cycle. This way, short therm biologic effect induced by zoledronic acid could be investigated. The authors found that a greater reduction in serum vascular endothelial growth factor (VEGF) occurred in the zoledronic acid group at day 5 than in the control group, although this effect could not be observed after day 21. Furthermore, the authors investigated serum reproductive hormones within the TGF-beta family (e.g., activin, TGF-beta-1, inhibin and follistatin) and observed that follistatin levels dropped more from baseline in postmenopausal zoledronic acid treated patients, which is interesting considering the still puzzling benefit of zoledronic acid in postmenopausal women. 

**Table 1:** Summary of neoadjuvant studies with chemotherapy in combination with zoledronic acid *.

Study	Intervention	Inclusion criteria	Primary endpoint	Secondary endpoints	(Estimated) enrollment	Estimated completed enrollment
NEOZOL	8 cycles CT only (first 4 cycles doxorubicin + cyclophosphamide, last 4 cycles docetaxel)	Breast cancer (TNM IIB, IIIa) 3 cm and largers in maximal diameter	Decrease in serum VEGF concentration treatment	Change in disseminated tumor cells in the bone marrow Change in serum markers of apoptosis Change in tumor markers of apoptosis and proliferation Assessment tumor response Change in circulating gamma-delta-T-cell activation	76	November 2013
	8 cycles CT with zoledronic acid (4 mg i.v.)					
					
					
					
ZoNantax	Cyclophosphamide, adriamycin (every 21 days for 4 cycles) Trastuzumab 8 mg/kg loading dose, then 3 times every 21 days for 3 cycles plus docetaxel 8 cycles with zoledronic acid (4 mg i.v.)	Stage IIA to IIIB HER-2 positive breast cancer	Residual cancer burden	Toxicity Difference in gene expression according to treatment response	56	November 2014
					
**Studies with completed accrual**						
Aft*et al.*	Neoadjuvant/adjuvant CT only (with four cycles of neoadjuvant epirubicin plus docetaxel and two cycles of adjuvant epirubicin and docetaxel)	Clinical stage II–III breast cancer	Number of patients with detectable DTCs at 3 months	Impact of zoledronic acid on relapse Effect of treatment on quality of life in	120	Completed
	Neoadjuvant/adjuvant CT in combination with zoledronic acid (4 mg i.v.) 3-weekly for 1 year					
NEOZOTAC	6 cycles CT only (docetaxel, adryamycin, cyclophosphamide)	T2 (≥2 cm and positive lymph nodes), T2 (≥3 cm), ≥ T3, T4, any N, M0 breast cancer	Pathologic complete response	Clinical response Tolerability Disease-free and overall survival Heterogeneity of ER/PR and HER2 measurement in core biopsy and surgical specimen Long-term outcome (disease free survival and overall survival)	250	Completed
	6 cycles CT only with zoledronic acid (4 mg i.v.)					
					
					
JONIE-1		Stage IIA, IIB, HER2-negative breast cancer	Pathologic complete response	Clinical response Disease free survival	188	Completed
					
ANZAC	6 cycles CT only	T2 breast tumor or above	Increase in apoptotic index between diagnostic core biopsy and repeat core biopsy	Reduction in Ki67 between preoperative biopsy and operative specimen Changes in serum angiogenesis markers Changes in bone biochemical markers Detection of and changes in circulating tumor cells in peripheral blood Prediction of pathological response by MRI calculated from the sequence of apparent diffusion coefficient	40	Completed
	6 cycles CT with zoledronic acid (4 mg i.v.) after first cycle chemotherapy only					
					
					
					

* Data obtained from [47].

## 7. Conclusions

In summary, there are several features of bisphosphonates which can contribute to an anti-tumor effect and can inhibit tumor growth. This given in combination with still sparse preclinical and clinical evidence for a benefit of neoadjuvant treatment, helps warranting clinical and translational research into this field. In the next few years response results and long-term outcome results of several neoadjuvant chemotherapy trials are expected. Translational research is represented in most of these trials. Hopefully, clinical and translational results will provide more answers to the question whether zoledronic acid in combination with chemotherapy can enhance tumor response.
